# Fatty Acids as Biomarkers of the Production Season of Caciocavallo Palermitano Cheese

**DOI:** 10.3390/ani11092675

**Published:** 2021-09-12

**Authors:** Giuseppe Maniaci, Antonino Di Grigoli, Adriana Bonanno, Cristina Giosuè, Vincenzo Ilardi, Marco Alabiso

**Affiliations:** 1 Department of Agricultural, Food and Forest Sciences (SAAF), University of Palermo, Viale Delle Scienze n. 13, 90128 Palermo, Italy; giuseppe.maniaci@unipa.it (G.M.); adriana.bonanno@unipa.it (A.B.); marco.alabiso@unipa.it (M.A.); 2 Institute for Anthropic Impacts and Sustainability in the Marine Environment, National Council of Research (IAS-CNR), Lungomare Cristoforo Colombo 4521, Loc. Addaura, 90149 Palermo, Italy; cristina.giosue@ias.cnr.it; 3 Department of Earth and Marine Sciences (DISTeM), University of Palermo, Via Archirafi 26, 90133 Palermo, Italy; vincenzo.ilardi@unipa.it

**Keywords:** autochthonous cow breed, pasture, production season, cheese, chemical composition, fatty acids

## Abstract

**Simple Summary:**

In the Mediterranean environment, climatic variability determines a discontinuity in the forage availability of pastures. Caciocavallo Palermitano is a cheese obtained from the milk of the Cinisara cattle breed, mainly raised on pasture. The present study investigated the fatty acid profile of cheeses produced in two typical farms in the four seasons of the year, with the aim of identifying specific fatty acids that can be used as biomarkers to discriminate the productions obtained in the different seasons, in order to economically enhance those seasonal productions that have better health characteristics. The results revealed the possibility of distinguishing spring productions from those of other seasons on the basis of the higher content of some fatty acids deriving from grazing fresh forage, the availability of which is greater in spring. Further studies should be conducted to also evaluate the possibility of using fatty acids as biomarkers of different diets.

**Abstract:**

This experiment aims to evaluate the potential of fatty acids (FA) of Caciocavallo Palermitano cheese as biomarkers of production season and pasture-based diet. A total of 48 cheeses were made in the four seasons with milk from two farms that raised cows of Cinisara breed. The animals were fed on pasture with supplementation of wheat bran and wheat straw in the barn, and in summer also with *Opuntia ficus-indica* cladodes. The chemical composition and FA profile of cheese were influenced by the season and not by the farm. In particular, cheeses produced in spring were characterized by higher protein and lower fat, and showed higher contents in trans-vaccenic acid, α-linolenic acid, rumenic acid, n-3 polyunsaturated FA (PUFA), and total PUFA. In winter, the lower availability of grazing forage, requiring a higher level of feeding integration, was responsible for an increase of saturated FA (SFA). The multivariate analysis distinguished clearly the cheeses made in winter and spring, while those produced in autumn and summer showed some overlapping points. Further investigations should be carried out to evaluate the effects of type and level of feeding integration on the presence of FA more suitable to be used as biomarkers of period and diet.

## 1. Introduction

The autochthonous breeds’ preservation is a strategic tool to maintain the history and the culture of particular habitats, agro-zootechnical systems, and local products that often are characterized by specific organoleptic and nutritional traits. Indeed, these breeds, as a result of natural selection, are adapted to their specific environments, producing also in harsh conditions where the specialized breeds fail to express their genetic potential [1].

Cinisara is a dual-purpose cow breed reared in Sicily, principally in the marginal areas located in Palermo, Messina, and Trapani provinces. Milk from Cinisara cows is mainly used to manufacture the Caciocavallo Palermitano cheese following a traditional method [2,3]. The economic income obtained from the sale of fresh meat is low, due to the competition with meat from specialized breeds. The products from processed meat, including bresaola and salami, could represent a considerable added value for the small farms of Cinisara breed, planning adequate promotion activities [4,5].

Caciocavallo Palermitano is a typical stretched-curd cheese, produced using whole and raw cow milk, characterized by a firm paste and a parallelepiped shape. During ripening, the color of paste changes from a distinctive straw to ochre. This cheese is historically linked to indigenous cow breeds, especially Cinisara, being able to exploit the natural pastures of the hills and semi-arid areas of Sicily [2]. Indeed, the cheese quality is the result of the interaction of different factors, such as the genetic characteristics of animals (breed) and the feeding system (fresh or preserved forage and the botanical composition of herbaceus vegetation at pasture), but also the microbial activity in milk and cheese, especially during ripening [2].

The diet is a very important factor affecting the fatty acid (FA) composition of dairy products. Different studies have shown that milk produced in grazing systems is characterized by an optimal FA profile from a human health point of view [6,7].

The intake of fresh forages, compared with diets based on dry forages and concentrates, increases the milk and cheese contents of n-3 polyunsaturated FA (PUFA), n-6 PUFA, and rumenic acid (RA, C18:2 c9t11), the main isomer of conjugated linoleic acids (CLAs) [8]. In these products, the CLA arises from both incomplete microbial hydrogenation of dietary PUFA in the rumen and, indirectly, from the activity of Δ-9 desaturase in mammary tissue that dehydrogenates the trans-vaccenic acid (TVA, 18:1 t11) produced in the rumen [9]. Thus, the RA concentration is mainly influenced by diet and, in particular, is higher in milk from animals fed pasture than from those fed dry diets, and decreases with the drying of the grass [10,11]. In this regard, the FA profile or specific PUFA content can be used to identify the type of feeding system [12], also in relation to the production period, and to detect the geographical origin of dairy products, a guarantee often required from protected designation of origin cheeses [13].

Indeed, even the grazing season can affect the FA profile of dairy products, considering the seasonal differences in grass availability and botanical composition of the pasture [11,14]. The FA profile depends also on the herbage phenological stage [15,16,17], as an increasing trend of α-linolenic acid (ALA, C18:3 n-3) and RA with herbage maturity was observed in milk from sheep grazing on different Mediterranean botanical species [18,19]. Moreover, grazing management and animal characteristics may also affect milk FA profile [14,20].

In the Mediterranean subtropical climate, the pastures start growing in autumn after the first rainfalls, with a winter slowdown and intense growth in spring when most of the plants are in the flowering phase; subsequently, the growth is interrupted by the hot and dry summer period [21].

However, little is known about how seasonal changes of fresh herbage phenology influence the FA profile of dairy products from native cows that are grazing all year around. In the farms rearing Cinisara breed, feeding is based on pasture all year, supplemented with concentrates, hay, and/or straw according to the forage availability and the productivity of cows. During the summer, *Opuntia ficus-indica* cladodes are also used as integration.

The aim of the present study was to evaluate the seasonal variation of FA profile in Caciocavallo Palermitano cheese manufactured in traditional Cinisara farms, also to identify specific PUFA suitable to be used as biomarkers of dairy products obtained in feeding systems and in production periods ensuring high pasture amounts for grazing cows.

## 2. Materials and Methods

### 2.1. Farms, Animals and Diet

In the typical production area of Caciocavallo Palermitano cheese, 48 samples of milk and cheeses were collected fortnightly in 2 farms (A and B, located in the Palermo area, in Sicily, Italy, from 400 to 600 m above sea level) over an entire production year (September–August). All cows present in both farms, equal to 55 Cinisara cows, were involved in this study, with calving distributed throughout the year, and reared according to traditional system based on pasture exploitation. The milking of the cows was carried out manually and in the presence of the calf, according to the system typically adopted for the autochthonous breeds. In particular, the animals used constantly the farm’s pasture, and were supplemented during the morning milking with wheat bran and wheat straw, and *Opuntia ficus-indica* cladodes in summer. The cheese was made only with the morning milk, using the afternoon one to feed the calves. Therefore, the management of cows was in full accordance to the Animal Welfare and Good Clinical Practice (Directive 2010/63/EU) and had approval of the local Bioethics Committee (protocol number: UNPA-CLE-Prot. 84097). The relevant farm characteristics and formulation of diets administrated indoor are reported in Table 1.

A total of 48 Caciocavallo Palermitano cheeses, equally distributed in the 4 seasons (autumn, winter, spring, and summer), were made according to the artisanal procedure using wooden tools as reported by Bonanno et al. [2]. The bulk milk of each farm was processed separately every 15 days in the same cheese factory. The cheeses (initial weight of about 7–8 kg) were dropped in a saturated brine for 7–8 d (1 day/kg) and aged for 2 months, at a temperature of 14–16 °C and at 75–85% of relative humidity.

### 2.2. Sampling and Analysis

The herbage of pasture was sampled monthly from five grazing areas (1.5 m × 1.5 m) randomly located in the pasture to determine the floristic composition. The grazing behavior of cows in the pasture was also observed and recorded monthly with regard to the selection of pabular essences. Based on these observations, samples of selected forage were recomposed by manual plucking of plant parts [22]. Samples of feeds and pabular essences selected at pasture were analyzed for dry matter (DM, method 967.03), crude protein (CP, N × 6.25) (method 988.05), ether extract (EE, method 920.29), and ash (method 942.05) contents, following the recommendations of the AOAC [23]. Neutral detergent fiber (NDF) was also determined [24].

Raw whole milk was sampled before processing and analyzed for fat, protein, casein, and lactose contents (CombiFoss 6000; Foss Electric A/S, Hillerød, Denmark).

For each 2-month aged cheese, DM, EE, CP (N × 6.38), and ash content were determined according to International Dairy Federation (IDF) standards [25,26,27,28], respectively.

Feeds and forages FA were extracted according to the method developed by O’Fallon et al. [29], with C23:0 as the internal standard (Sigma-Aldrich, Milano, Italy), and were identified using the procedure described below for cheese FA.

FA in lyophilized cheese samples (100 mg) were directly methylated with 2 mL of 0.5 M NaOCH_3_ at 50 °C for 15 min, followed by 1 mL of 5% HCl in methanol at 50 °C for 15 min as described by Bonanno et al. [30]. Fatty acid methyl esters (FAME) were recovered in hexane (1.5 mL). Each sample (1 μL) was injected by autosampler into an HP 6890 gas chromatography system equipped with a flame ionization detector (Agilent Technologies Inc., Santa Clara, CA, USA). The separation and identification of each FA was performed as described by Alabiso et al. [31].

The health-promoting index (HPI) [(n-3 PUFA + n-6 PUFA + MUFA)/(C12:0 + (4 × C14:0) + C16:0)] and thrombogenic index (TI) [(C14:0 + C16:0 + C18:0)/((0.5 × Σ MUFA) + (0.5 × Σ n-6 PUFA) + (3 × Σ n-3 PUFA) + (n-3/n-6))] were calculated, respectively, as suggested by Ashkezary et al. [32] and Ulbricth and Southgate [33].

### 2.3. Statistical Analysis

The data were subjected to statistical analysis using the SAS 9.2 software [34]. Chemical traits and FA composition were analyzed according to a mixed model including the fixed effects of production season (S, with four levels: autumn, winter, spring, and summer) and farm (F, with two levels), and the month of production as a random effect. The interaction S × F was removed from the model since it was not significant. Tukey’s test was used to compare means when the S effect was significant (*p* ≤ 0.05). The following intervals were used to define the production season: autumn (September, October, and November), winter (December, January, and February), spring (March, April, and May) and summer (June, July, and August).

To evaluate the specific contribution of the chemical traits and composition of FA in explaining the differences between cheeses due to the different production season, a principal component analysis (PCA) was carried out, with the PRINCOMP SAS procedure. The variables used in the analysis were standardized by multiplying them by the inverse of the standard deviation (1/SD) and identified by gradual selection with the STEPDISC SAS procedure. The selection of the main components was carried out according to the Kaiser method, keeping those with Eigen values higher than 1.00.

## 3. Results and Discussion

### 3.1. Pasture Composition

The two farms belonged to a similar pedo-climatic environment; therefore, the respective pastures were similar in relation to botanical composition and, consequently, treated as a single unit. With regard to the floristic composition, a total of 377 specific and infraspecific taxa were recognized, divided into 217 genera and 55 families. On average, each family contributed as following: Fabaceae (19%), Asteraceae (14%), Poaceae (12%), Apiaceae (5%), Brassicaceae (4%), Malvaceae (2%), and other (44%).

The chemical composition of the ingested fraction at pasture and feeds is shown in Table 2. As expected, passing from autumn to summer, DM strongly increased and CP decreased. Moreover, in summer, the ingested forage was higher in NDF and EE, as well as in linoleic acid (LA, C18:2 n-6), presumably due to the presence of seeds in the forage selected by the cows.

### 3.2. Milk and Cheese Composition

The daily milk yield and chemical composition of milk and cheese in relation to the production season are reported in Table 3. On the whole, there was a significant effect of production season, whereas no differences were observed between the farms.

In summer, corresponding to a lower quantity and quality of pasture, the daily milk yield was lower than in the other seasons.

In general, the percentage of milk fat was low, probably due to the milking system adopted mainly for autochthonous breeds, which, occurring in the presence of the calf, does not allow a complete release of the milk. The milk produced in spring and summer showed lower fat content than in the other seasons. Protein and casein contents were also lower in summer than in the other seasons. The cheese yield at 24 h was lower in spring and summer than in autumn and winter. The milk protein content was on average similar to the value reported by Altomonte et al. [35], while the fat was lower, probably due to the higher milk production levels registered in these cows.

The cheese composition was similar to those reported by Bonanno et al. [36] for the same product, made using milk of cows pasture-fed with the integration of hay and concentrate in the barn.

The production season affected significantly the chemical composition of cheese, except for DM. The protein and fat contents showed an opposite trend since protein was higher and fat was lower in spring. Similarly to what was found in milk, the protein content was higher in winter and spring than in summer and autumn. However, other authors have not found the same correspondence between the protein content of milk and that of cheeses, produced with animals raised on pasture and with the same cheese-making process [37].

The FA composition of Caciocavallo Palermitano cheese was mainly affected by production season, but not by the farm.

The FA profiles of cheeses in relation to the production season are reported in Table 4 and Table 5. On average, the FA profiles were comparable to those reported for Caciocavallo Palermitano cheeses by Bonanno et al. [2], although the latter showed a lower content in n-6 PUFA.

In spring, when the animals had presumably a greater intake of grazed forage than in autumn and winter, the butyric acid (C4:0) content was lower (*p* ≤ 0.05), as well as the other short- and medium-chain FA that are synthetized de novo in the mammary gland, even if at a not significant level. Milk from grazing animals may show a generalized reduction of de novo FA; indeed, the high level of dietary PUFA from pasture could compete with de novo FA for esterification in the mammary gland and thus decrease the synthesis of short- and medium-chain FA [38]. Moreover, in lactating cows a negative energy balance can occur at pasture, reducing the synthesis of short- and medium-chain FA in the mammary gland [37]. Feeding integration, provided in the season of pasture shortage, may have contributed to mitigate the general reduction of shorts and medium-chain FA, as found by Bargo et al. [39].

Cheeses made in winter were characterized by a higher myristic acid (C14:0) content than in the other seasons (*p* ≤ 0.01), and a higher palmitic acid (C16:0) content than in summer (*p* ≤ 0.01). Among SFA, C12:0, C14:0, and C16:0 are considered pro-atherogenic, while C18:0 is transformed into oleic acid (OA, C18:1 n-9) in the body tissues and has a hypocholesterolemic action [40]. In winter, greater feeding integration in the barn led to an increase in C14:0 and C16:0 as found in other investigations [11,12,37]. In spring and summer, C18:0 content was higher than in winter (*p* ≤ 0.01), as a consequence of less abundant feeding in the barn, as found by Coppa et al. [41] on milk from cows fed with different grazing systems compared to those fed exclusively in barns. Arachidic acid (C20:0) content was higher in summer than in other seasons (*p* ≤ 0.01), probably for the use of the cladodes of *Opuntia ficus-indica,* which, as it is reported in Table 2, has a good content of C20:0, in line with Andreu-Coll et al. [42].

The MUFA profile (Table 4) was influenced by the production season; in particular, C14:1 was higher in winter than in summer (*p* ≤ 0.01), contrary to C17:1, which was lower in winter than in summer (*p* ≤ 0.05). The C16:1 content was higher in spring than in winter (*p* ≤ 0.05), while the OA content was higher in summer than in winter (*p* ≤ 0.01) and spring (*p* ≤ 0.05). Finally, the contents of TVA and of the other C18:1 were higher in spring. The use of *Opuntia ficus-indica* cladodes could have increased the level of OA and total MUFA (Table 5), as found in other studies that have compared different levels of integration or the use of cladodes [11,12,37,43].

The higher levels of grazing intake favored the content of RA and other CLA isomers. The positive impact of grazing on the contents of TVA and RA in cheese is mainly due to the high amount of ALA in fresh forage. ALA is partially converted into TVA by the biohydrogenation process occurring in the rumen, whereas, in the mammary gland, TVA is converted to RA by the enzymatic oxidation operated by the delta-9 desaturase [44]. A similar trend was recorded for ALA and γ-linolenic acid (GLA) in relation to their highest content in spring (*p* ≤ 0.01), due to the positive effect of the increase in the intake of fresh forage at pasture [8].

The FA profile and health indexes in relation to the production season are reported in Table 5. The total FA content (% DM) was lower in spring than in other seasons, according to the lower fat level.

In winter, when the cows received higher indoor supplementation, the SFA content was higher than in summer (*p* ≤ 0.01), autumn, and spring (*p* ≤ 0.05). A higher SFA content in winter has been observed in other investigations [11,45] and, as reported by Ferlay et al. [46], is linked to the increase in hay and concentrate in the diet.

Cheese obtained from winter milk was lower in MUFA than were summer (*p* ≤ 0.01) and autumn (*p* ≤ 0.05) cheeses, as found in previous investigations [11,47].

The PUFA content was higher in spring than in summer (*p* ≤ 0.05), autumn, and winter (*p* ≤ 0.01), due to the increased intake of fresh pasture, as found by Romanzin et al. [48]; indeed, numerous studies report a “seasonal” effect on the FA composition of dairy products, with an increase of the concentration of PUFA and CLA in spring, due to the corresponding gain in the content of PUFA in fresh forage [8,49]. As reported by several authors, the interaction between forage and concentrate (substitution effect) leads to a different use of forage depending on the quantity and quality of both feeding sources, with a proportional reduction in the intake of forage to the increase in concentrates, especially at high levels of input in the barn [50,51]. It is reasonable that in spring, with a lower level of concentrate and straw, the cows have ingested a higher amount of herbage at pasture, whereas in summer, the integration with the cladodes of *Opuntia ficus-indica* could may have compensated for the low intake of fresh forage, lacking at pasture in the summer season.

It should be emphasized that in summer, despite the decline of FA profile that characterized the pastures grazed in this season (Table 2), the FA profile of the cheeses remained at the same quality levels recorded for autumn and winter; probably, the administration of *Opuntia ficus-indica* cladodes helped to limit the effect of pasture FA profile by avoiding the increase in SFA in favor of MUFA and PUFA.

Among the health indices investigated, the MUFA/SFA and PUFA/SFA ratios, n-3 PUFA, HPI, and TI were influenced by the season. Due to the higher MUFA content and the lower SFA content of summer cheeses, the MUFA/SFA ratio was higher in summer than in winter (*p* ≤ 0.01); in addition, differences were found between autumn and winter (*p* ≤ 0.05).

The PUFA/SFA index is used to assess the impact of diet on cardiovascular health (CVH), hypothesizing that PUFA in the diet depress low-density lipoprotein cholesterol (LDL-C); thus, the higher this ratio, the more positive the effect. The values obtained were on average low and comparable to those found by Bonanno et al. [19] in cheeses obtained from the milk of sheep grazing on ryegrass. In spring, the higher PUFA content resulted in a higher PUFA/SFA ratio than in winter (*p* ≤ 0.05).

The higher intake of grazing forage in spring, rich in PUFA, resulted in a higher n-3 PUFA content than in the other seasons (*p* ≤ 0.01), due to the contribution of ALA.

The HPI index is currently used for dairy products, and its high values indicate a more beneficial fat to human health. HPI was higher in summer and autumn than in winter and spring (*p* ≤ 0.01), and higher than that found on Caciocavallo Palermitano by Bonanno et al. [2].

Cheeses produced in winter showed a higher TI than those produced in spring, summer, and autumn (*p* ≤ 0.001). The TI index characterizes the thrombogenic potential of FA, indicating the tendency to form clots in blood vessels, put in relationship to the pro-thrombogenic FA (C12:0, C14:0, and C16:0) and the anti-thrombogenic FA (MUFA, n-3 PUFA, and n-6 PUFA). Therefore, the consumption of foods or products with a lower TI is beneficial for CVH, though no organization has yet provided the recommended values for TI [52].

**Figure 1:**
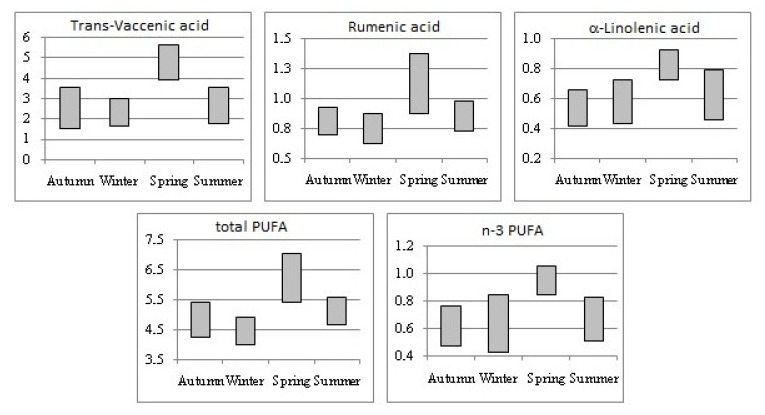
Seasonal variation (minimum value–maximum value) in fatty acids (g/100 g FA) in cheeses.

From the examination of Figure 1, which shows the variation of seasonal levels for some cheese FA, it emerges that these FA were singularly effective in differentiating the cheeses according to the season; indeed, a complete distinction of cheese produced in spring, from milk of cows fed with pasture-based diet, was obtained for the levels of TVA, RA, and ALA that, together with the n-3 PUFA and total PUFA, have revealed their potential to be used biomarkers of production season or feeding regime for dairy products obtained from milk of autochthonous breeds whose diet is mainly based on grazing fresh forage at pasture.

### 3.3. Multivariate Analysis

**Figure 2:**
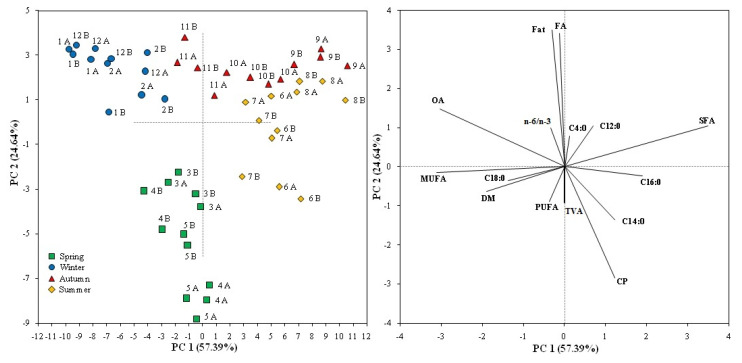
PCA analysis based on chemical traits of milk and cheese and FA composition for production month. The length of each vector is proportional to its contribution to the main components. Abbreviations: DM = dry matter; CP = crude protein; FA = total fatty acids; SFA = saturated fatty acids; MUFA = monounsaturated fatty acids; PUFA = polyunsaturated fatty acids; OA = oleic acid; TVA = trans-vaccenic acid; 1 to 12 = months; A and B = farms.

The plot generated by PCA is shown in Figure 2. The length of each vector measures the contribution of each selected variable on the main components.

The first two principal components accounted for 82.03% of the total variance, allowing to discriminate the cheeses of the different production seasons. In particular, the first principal component (57.39% of the total variance) was able to discriminate the winter cheese from those produced in autumn and summer on the basis of the contributions of SFA, C16:0, OA, MUFA, and, to a lesser extent, DM, C12:0, and C14:0. Instead, the second principal component (24.64% of the total variance) was responsible for the separation of spring cheeses from those produced in the other seasons, especially due to the contribution of CP, fat, total FA, PUFA, TVA and n-6/n-3 ratio. A minor differentiation occurred between the cheeses produced in autumn and summer, evidenced by the partial overlap in the first quadrant of the Cartesian plane.

The separation generated by the PCA is in line with the differentiation among cheeses due to the production season; indeed, the main components that had a greater weight in the discrimination of cheeses are those that were significantly different in the statistical analysis.

## 4. Conclusions

Caciocavallo Palermitano cheese showed differences in chemical composition and FA profile determined by the different production season. The relationship between the level of feeding supplements provided indoor, the grazing intake, and the phenological stage of the pabular essences in the different seasons contributed to confer specific characteristics to the dairy products. In particular, cheeses produced in spring were characterized by higher protein and lower fat contents, and a FA profile with a higher content in TVA, ALA, RA, n-3 PUFA, and total PUFA due to the effect of pasture-based diet favoring the transfer of PUFA beneficial for human health from fresh forage to the final dairy products, although HPI was lower than in summer and autumn; accordingly, these FA can be considered among potential biomarkers of dairy products obtained in spring, in contrast to other seasons where no clear distinction was found between FA. During winter and autumn, the lower availability of grazing forage, requiring a higher level of feeding integration in the barn, resulted in an increase of SFA content in the cheese. In summer, the administration of *Opuntia ficus-indica* cladodes seems to have contributed to limit the increase of SFA in favor of MUFA and PUFA, mitigating the effect of the pasture decline commonly observed in this season in the production area of this cheese. The multivariate analysis distinguished clearly the cheeses produced in winter and spring, while the cheeses produced in autumn and summer, although distinct, had some overlapping points. Further investigations should be carried out to evaluate the effects of feeding supplements, provided indoor to cows to integrate the scarce grazing resources, on the presence of FA more suitable to be used as biomarkers of production period and pasture-based diet.

## Figures and Tables

**Table 1:** Farm characteristics and diet formulation.

Season	Autumn		Winter		Spring		Summer	
Farm	A	B	A	B	A	B	A	B
Farm characteristics								
Available grazing areas, ha	26	30	26	30	26	30	26	30
Lactating cows, n.	15	17	18	25	18	26	10	15
Indoor diet ingredients, kg/animal per day								
Wheat bran	7.0	7.0	7.8	7.9	4.9	4.9	5.6	5.0
Wheat straw	4.0	4.0	4.0	4.0	4.0	4.0	4.0	4.0
*Opuntia ficus-indica* cladodes							9.0	11.0

**Table 2:** Chemical composition (% DM) and fatty acids profile (g/100 g FA) of the ingested fraction of grazing pastures and feeds provided indoor.

	Pastures								Feed		
Season	Autumn		Winter		Spring		Summer		Wheat Bran	Wheat Straw	Cladodes ^1^
Farm	A	B	A	B	A	B	A	B			
Dry Matter, %	19.16	18.70	16.26	15.52	18.79	19.66	78.15	76.32	89.54	92.71	8.64
Crude Protein	19.44	23.37	21.32	23.75	17.93	16.12	9.94	9.87	18.11	6.37	8.31
Ether extract	3.62	4.30	5.00	4.44	3.87	3.35	8.81	7.07	4.65	1.73	2.41
Ash	11.86	14.75	12.40	13.87	9.32	10.11	8.94	10.58	4.39	6.96	27.35
NDF	49.71	47.88	38.45	35.61	44.48	40.89	51.59	53.27	26.22	73.22	31.08
Total FA, % DM	3.23	3.85	4.50	4.22	3.44	2.85	7.98	6.41	3.86	1.56	2.15
C12:0	0.53	0.49	0.57	0.53	0.44	0.42	0.41	0.56	0.00	2.14	0.00
C14:0	2.17	2.23	2.21	2.61	1.80	1.91	0.85	0.73	10.37	4.55	0.00
C14:1	0.38	0.37	0.41	0.4	0.35	0.31	0.3	0.31	0.00	0.00	0.00
C16:0	17.60	17.70	15.62	15.32	20.1	21.54	27.71	27.92	14.45	32.74	17.43
C16:1	1.40	1.51	1.45	1.66	1.46	1.05	1.41	1.41	0.21	0.00	0.00
C18:0	2.38	2.43	1.96	1.76	3.58	2.79	6.90	7.01	0.79	18.10	5.04
C18:1 c9	2.79	2.95	2.25	2.75	3.17	3.01	18.04	17.85	16.65	7.52	11.14
C18:2 n-6	15.77	15.81	12.21	12.01	22.70	20.95	35.18	35.02	51.71	11.31	35.40
C18:3 n-3	56.75	56.91	62.79	62.73	46.30	47.90	9.15	9.19	5.81	23.64	16.20
C20:0	0.00	0.00	0.00	0.00	0.00	0.00	0.00	0.00	0.00	0.00	14.70

^1^ Opuntia ficus-indica cladodes

**Table 3:** Daily milk yield and chemical composition of raw milk (%) and Caciocavallo Palermitano cheese (% DM) in relation to the production season.

	Season ^1^				SEM ^2^	Significance
Parameters	Autumn	Winter	Spring	Summer		Season
Milk						
Daily yield, kg/head	11.7 ^a^	11.5 ^a^	12.7 ^a^	9.8 ^b^	0.635	0.035
Fat	3.42 ^ABa^	3.52 ^Aa^	3.12 ^Bb^	3.14 ^Bb^	0.081	0.005
Protein	3.46 ^ABb^	3.70 ^Aa^	3.59 ^Aab^	3.27 ^Bc^	0.063	0.002
Casein	2.93 ^ABb^	3.22 ^Aa^	2.94 ^Ab^	2.66 ^Bc^	0.073	0.001
Lactose	4.60 ^b^	4.73 ^a^	4.60 ^b^	4.65 ^ab^	0.029	0.024
Cheese						
Yield at 24 h, kg/100 kg	11.4 ^ABa^	11.8 ^Aa^	10.8 ^BCb^	10.2 ^Cb^	0.195	<0.001
Dry Matter, %	65.3	65.1	67.5	70.4	1.543	0.096
Fat	45.3 ^Aa^	44.9 ^ABa^	41.0 ^Bb^	44.2 ^ABa^	0.749	0.002
Protein	44.6 ^Bb^	46.7 ^ABab^	48.4 ^Aa^	45.1 ^ABb^	0.856	0.007
Ash	8.02 ^a^	6.46 ^b^	7.89 ^b^	7.84 ^b^	0.308	0.017

The results indicate mean values of three measurements performed on each cheese. ^1^ Season: autumn (September, October, and November), winter (December, January, and February), spring (March, April, and May) and summer (June, July, and August). ^2^ SEM = standard error of the mean. On horizontal rows: A, B, and C = *p* ≤ 0.01, a, b, and c = *p* ≤ 0.05.

**Table 4:** Fatty acid profile (g/100 g FA) of Caciocavallo Palermitano cheese in relation to production season.

	Season ^1^				SEM ^2^	Significance
Parameters	Autumn	Winter	Spring	Summer		Season
C4:0	2.54 ^a^	2.54 ^a^	1.59 ^b^	2.29 ^ab^	0.249	0.019
C6:0	2.03	2.32	1.76	1.80	0.146	0.072
C8:0	1.27	1.58	0.81	1.11	0.252	0.214
C10:0	2.58	3.41	2.36	2.21	0.327	0.131
C12:0	2.93	3.92	2.13	2.46	0.572	0.214
C14:0	11.0 ^B^	12.9 ^A^	10.9 ^B^	10.4 ^B^	0.457	<0.001
C14:1	0.96 ^AB^	1.12 ^A^	0.93 ^AB^	0.76 ^B^	0.062	0.012
C15:0iso	0.34	0.32	0.32	0.32	0.009	0.385
C15:0	1.19	1.24	1.35	1.26	0.047	0.073
C16:0iso	0.32	0.30	0.31	0.28	0.024	0.638
C16:0	28.4 ^AB^	31.7 ^A^	29.0 ^AB^	27.0 ^B^	0.789	0.011
C16:1	0.22 ^ab^	0.19 ^b^	0.33 ^a^	0.24 ^ab^	0.034	0.025
C17:0iso	0.51 ^ab^	0.45 ^b^	0.54 ^a^	0.54 ^a^	0.021	0.047
C17:0anteiso	1.83	1.86	1.68	1.82	0.080	0.303
C17:0	0.72	0.72	0.79	0.79	0.019	0.060
C17:1	0.25 ^ab^	0.22 ^b^	0.25 ^ab^	0.29 ^a^	0.015	0.007
C18:0	10.3 ^AB^	8.56 ^B^	12.4 ^A^	12.2 ^A^	0.530	0.003
C18:1 c9 OA	22.9 ^ABab^	18.4 ^Bc^	19.8 ^Bbc^	24.2 ^Aa^	0.981	0.002
C18:1 t11 TVA	3.15 ^B^	2.82 ^B^	4.14 ^A^	3.09 ^B^	0.218	<0.001
Other C18:1	1.37 ^ABb^	0.89 ^Bb^	2.12 ^Aa^	1.39 ^ABb^	0.174	<0.001
Other C18:2	0.75	0.62	0.81	0.60	0.092	0.070
C18:2 n-6 LA	2.18	2.01	2.52	2.54	0.179	0.143
CLA C18:2 c9t11 RA	0.93 ^b^	0.74 ^b^	1.24 ^a^	0.82 ^b^	0.083	0.010
Other CLA ^c^ isomers	0.10 ^AB^	0.04 ^B^	0.13 ^A^	0.06 ^AB^	0.021	0.011
C18:3 n-3 ALA	0.55 ^B^	0.50 ^B^	0.84 ^A^	0.58 ^B^	0.049	<0.001
C18:3 n-6 GLA	0.15 ^ABb^	0.12 ^Bb^	0.18 ^Aa^	0.13 ^Bb^	0.007	<0.001
C20:0	0.19 ^B^	0.16 ^B^	0.20 ^B^	0.27 ^A^	0.010	<0.001
C20:2 n-6	0.04	0.03	0.04	0.05	0.010	0.719
C20:3 n-6	0.07	0.07	0.10	0.08	0.008	0.078
C20:4 n-6 AA	0.14	0.14	0.15	0.15	0.008	0.439
C20:5 n-3 EPA	0.02	0.02	0.05	0.05	0.008	0.099
C22:0	0.07	0.06	0.08	0.08	0.008	0.059
C22:5 n-3 DPA	0.04	0.05	0.08	0.07	0.012	0.070

The results indicate mean values of three measurements performed on each of cheese. ^1^ Season: autumn (September, October, and November), winter (December, January, and February), spring (March, April, and May) and summer (June, July, and August). ^2^ SEM = standard error of the means. On horizontal rows: A and B = *p* ≤ 0.01, a, b, and c = *p* ≤ 0.05. OA = oleic acid; TVA = trans-vaccenic acid; LA = linoleic acid; ALA = α-linolenic acid; GLA = γ-linolenic acid; RA = rumenic acid; CLA = conjugated linoleic acid; AA = arachidonic acid; EPA = eicosapentaenoic acid; DPA = docosapentaenoic acid.

**Table 5:** Fatty acid profile (g/100 g FA) and health indexes of Caciocavallo Palermitano cheese in relation to production season.

	Season ^1^				SEM ^2^	Significance
Parameters	Autumn	Winter	Spring	Summer		Season
Total FA, % DM	41.0 ^Aa^	40.6 ^ABa^	37.0 ^Bb^	40.0 ^ABa^	0.711	<0.001
SFA ^3^	66.2 ^ABb^	72.1 ^Aa^	66.3 ^ABb^	64.9 ^Bb^	1.166	0.006
MUFA ^4^	28.8 ^ABa^	23.6 ^Bb^	27.5 ^ABab^	30.0 ^Aa^	0.984	0.004
PUFA ^5^	4.94 ^Bb^	4.31 ^Bb^	6.19 ^Aa^	5.16 ^ABb^	0.242	<0.001
MUFA/SFA	0.44 ^ABa^	0.33 ^Bb^	0.42 ^ABab^	0.46 ^Aa^	0.022	0.007
PUFA/SFA	0.07 ^ab^	0.06 ^b^	0.09 ^a^	0.08 ^ab^	0.005	0.021
n-6	2.57	2.37	2.94	2.99	0.200	0.134
n-3	0.59 ^B^	0.56 ^B^	0.97 ^A^	0.70 ^B^	0.053	<0.001
n-6/n-3	4.46	4.38	3.06	4.44	0.477	0.071
HPI ^6^	0.44 ^Aa^	0.32 ^Bc^	0.38 ^Bb^	0.47 ^Aa^	0.028	0.002
TI ^7^	2.79 ^B^	3.42 ^A^	2.84 ^B^	2.67 ^B^	0.091	<0.001

The results indicate mean values of three measurements performed on each of cheese. ^1^ Season: autumn (September, October, and November), winter (December, January, and February), spring (March, April, and May) and summer (June, July, and August). ^2^ SEM = standard error of the means. On horizontal rows: A and B = *p* ≤ 0.01, a, b, and c = *p* ≤ 0.05; ^3^ SFA = saturated fatty acids; ^4^ MUFA = monounsaturated fatty acids; ^5^ PUFA = polyunsaturated fatty acids; ^6^ HPI = health-promoting index; ^7^ TI = thrombogenic index.

## Data Availability

All data included in this study are available upon request by contacting the corresponding author.

## References

[1] Ciotola F., Albarella S., Liotta L., Contessa A., Di Meo G.P., Barbieri V., Peretti V. (2009). Native cattle breeds of Italy: Karyological profile. Ital. J. Anim. Sci..

[2] Bonanno A., Tornambè G., Bellina V., De Pasquale C., Mazza F., Maniaci G., Di Grigoli A. (2013). Effect of farming system and cheese making technology on the physicochemical characteristics, fatty acid profile, and sensory properties of Caciocavallo Palermitano cheese. J. Dairy Sci..

[3] Di Gregorio P., Di Grigoli A., Di Trana A., Alabiso M., Maniaci G., Rando A., Vallucci C., Finizio D., Bonanno A. (2017). Effects of different genotypes at the CSN3 and LGB loci on milk and cheese-making characteristics of the bovine Cinisara breed. Int. Dairy J..

[4] Maniaci G., Alabiso M., Francesca N., Giosuè C., Di Grigoli A., Corona O., Cardamone C., Graci G., Portolano B., Bonanno A. (2020). Bresaola made from Cinisara cattle: Effect of muscle type and animal category on physicochemical and sensory traits. CyTA J. Food.

[5] Alabiso M., Maniaci G., Giosuè C., Di Grigoli A., Bonanno A. (2021). Fatty Acid Composition of Salami Made by Meat from Different Commercial Categories of Indigenous Dairy Cattle. Animals.

[6] Coppa M., Gorlier A., Lonati M., Martin B., Russo E.M., Lombardi G. (2012). The management of the transition from hay to pasture-based diets affects milk fatty acid kinetics. Dairy Sci. Technol..

[7] Chilliard Y., Glasser F., Ferlay A., Bernard L., Rouel J., Doreau M. (2007). Diet, rumen biohydrogenation and nutritional quality of cow and goat milk fat. Eur. J. Lipid Sci. Technol..

[8] Nudda A., Battacone G., Boaventura Neto O., Cannas A., Francesconi A.H.D., Atzori A.S., Pulina G. (2014). Feeding strategies to design the fatty acid profile of sheep milk and cheese. R. Bras. Zootec..

[9] Griinari J.M., Corl B.A., Lacy S.H., Chouinard P.Y., Nurmela K.V.V., Bauman D.E. (2000). Conjugated linoleic acid is synthesized endogenously in lactating dairy cows by Δ9-desaturase. J. Nutr..

[10] Prandini A., Sigolo S., Cerioli C., Piva G. (2009). Survey on conjugated linoleic acid (CLA) content and fatty acid composition of Grana Padano cheese produced in different seasons and areas. Ital. J. Anim. Sci..

[11] Chion A.R., Tabacco E., Giaccone D., Peiretti P.G., Battelli G., Borreani G. (2010). Variation of fatty acid and terpene profiles in mountain milk and “Toma piemontese” cheese as affected by diet composition in different seasons. Food Chem..

[12] Agradi S., Curone G., Negroni D., Vigo D., Brecchia G., Bronzo V., Panseri S., Chiesa L.M., Peric T., Danes D. (2020). Determination of fatty acids profile in Original Brown cows dairy products and relationship with alpine pasture farming system. Animals.

[13] Caredda M., Addis M., Ibba I., Leardi R., Scintu M.F., Piredda G., Sanna G. (2017). Building of prediction models by using Mid-Infrared spectroscopy and fatty acid profile to discriminate the geographical origin of sheep milk. LWT.

[14] Coppa M., Farruggia A., Ravaglia P., Pomiès D., Borreani G., Le Morvan A., Ferlay A. (2015). Frequent moving of grazing dairy cows to new paddocks increases the variability of milk fatty acid composition. Animal.

[15] Buccioni A., Rapaccini S., Antongiovanni M., Minieri S., Conte G., Mele M. (2010). Conjugated linoleic acid and C18:1 isomers content in milk fat of sheep and their transfer to Pecorino Toscano cheese. Int. Dairy J..

[16] Khan N.A., Cone J.W., Fievez V., Hendriks W.H. (2012). Causes of variation in fatty acid content and composition in grass and maize silages. Anim. Feed Sci. Technol..

[17] Glasser F., Doreau M., Maxin G., Baumont R. (2013). Fat and fatty acid content and composition of forages: A meta-analysis. Anim. Feed Sci. Technol..

[18] Cabiddu A., Decandia M., Addis M., Piredda G., Pirisi A., Molle G. (2005). Managing Mediterranean pastures in order to enhance the level of beneficial fatty acids in sheep milk. Small Rumin. Res..

[19] Bonanno A., Di Grigoli A., Mazza F., De Pasquale C., Giosuè C., Vitale F., Alabiso M. (2016). Effects of ewes grazing sulla or ryegrass pasture for different daily durations on forage intake, milk production and fatty acid composition of cheese. Animal.

[20] Couvreur S., Hurtaud C., Lopez C., Delaby L., Peyraud J.L. (2006). The linear relationship between the proportion of fresh grass in the cow diet, milk fatty acid composition, and butter properties. J. Dairy Sci..

[21] Marino V.M., Schadt I., Carpino S., Caccamo M., La Terra S., Guardiano C., Licitra G. (2014). Effect of Sicilian pasture feeding management on content of α-tocopherol and β-carotene in cow milk. J. Dairy Sci..

[22] Di Grigoli A., Di Trana A., Alabiso M., Maniaci G., Giorgio D., Bonanno A. (2019). Effects of grazing on the behaviour, oxidative and immune status, and production of organic dairy cows. Animals.

[23] (2012). Official Methods of Analysis of Aoac International.

[24] Van Soest P.J., Robertson J.B., Lewis B.A. (1991). Methods for dietary fiber, neutral detergent fiber, and non-starch polysaccharides in relation to animal nutrition. J. Dairy Sci..

[25] IDF (International Dairy Federation) (1982). Cheese and Processed Cheese Product. Determination of the Total Solids Content.

[26] IDF (International Dairy Federation) (1986). Cheese and Processed Cheese Product. Determination of Fat Content-Gravimetric Method (Reference Method).

[27] IDF (International Dairy Federation) (1964). Determination of the Protein Content of Processed Cheese Products.

[28] IDF (International Dairy Federation) (1964). Determination of the Ash Content of Processed Cheese Products.

[29] O’Fallon J.V., Busboom J.R., Nelson M.L., Gaskins C.T. (2007). A direct method for fatty acid methyl ester synthesis: Application to wet meat tissues, oils, and feedstuffs. J. Anim. Sci..

[30] Bonanno A., Di Grigoli A., Vitale F., Alabiso M., Giosuè C., Mazza F., Todaro M. (2016). Legume grain-based supplements in dairy sheep diet: Effects on milk yield, composition and fatty acid profile. Anim. Prod. Sci..

[31] Alabiso M., Maniaci G., Giosuè C., Gaglio R., Francesca N., Di Grigoli A., Portolano B., Bonanno A. (2020). Effect of muscle type and animal category on fatty acid composition of bresaola made from meat of Cinisara cattle: Preliminary investigation. CyTA-J. Food.

[32] Ashkezary M.R., Bonanno A., Todaro M., Settanni L., Gaglio R., Todaro A., Alabiso M., Maniaci G., Mazza F., Di Grigoli A. (2020). Effects of adding solid and molten chocolate on the physicochemical, antioxidant, microbiological, and sensory properties of ewe’s milk cheese. J. Food Sci..

[33] Ulbricth T.L.V., Southgate D.A.T. (1991). Coronary Heart Disease, Seven Dietary Factors. Lancet.

[34] (2010). AS/STAT Qualification Tools User’s Guide in SAS 9.

[35] Altomonte I., Salari F., Neglia A., Martini M. (2016). Milk yield and quality characteristics of Cinisara and Modicana cows reared on a farm in the province of Palermo (Sicily-Italy). Large Anim. Rev..

[36] Bonanno A., Di Grigoli A., Tornambè G., Formoso B., Alicata M.L., Procida G., Pizzoferrato L. Effects of feeding on nutritional and aromatic characteristics of caciocavallo palermitano cheese. Proceedings of the 6th International Meeting on Mountain Cheese.

[37] Todaro M., Bonanno A., Scatassa M.L. (2014). The quality of Valle del Belice sheep’s milk and cheese produced in the hot summer season in Sicily. Dairy Sci. Technol..

[38] Esposito G., Masucci F., Napolitano F., Braghieri A., Romano R., Manzo N., Di Francia A. (2014). Fatty acid and sensory profiles of Caciocavallo cheese as affected by management system. J. Dairy Sci..

[39] Bargo F., Delahoy J.E., Schroeder G.F., Muller L.D. (2006). Milk fatty acid composition of dairy cows grazing at two pasture allowances and supplemented with different levels and sources of concentrate. Anim. Feed Sci. Technol..

[40] Sinclair A.J. (1993). Dietary fat and cardiovascular disease: The significance of recent developments for the food industry. Food Aust..

[41] Coppa M., Ferlay A., Monsallier F., Verdier-Metz I., Pradel P., Didienne R., Farruggia A., Montel M.C., Martin B. (2011). Milk fatty acid composition and cheese texture and appearance from cows fed hay or different grazing systems on upland pastures. J. Dairy Sci..

[42] Andreu-Coll L., Cano-Lamadrid M., Sendra E., Carbonell-Barrachina A., Legua P., Hernández F. (2019). Fatty acid profile of fruits (pulp and peel) and cladodes (young and old) of prickly pear *Opuntia ficus-indica* (L.) Mill. from six Spanish cultivars. J. Food Compost. Anal..

[43] El Otmani S., Chebli Y., Chentouf M., Hornick J.L., Cabaraux J.F. (2021). Effects of Olive Cake and Cactus Cladodes as Alternative Feed Resources on Goat Milk Production and Quality. Agriculture.

[44] Szumacher-Strabel M., Cieslak A., Nowakowska A. (2009). Effect of oils rich in linoleic acid on in vitro rumen fermentation parameters of sheep, goats and dairy cows. J. Anim. Feed Sci..

[45] Collomb M., Bisig W., Butikofer U., Sieber R., Bregy M., Etter L. (2008). Fatty acid composition of mountain milk from Switzerland: Comparison of organic and integrated farming systems. Int. Dairy J..

[46] Ferlay A., Martin B., Pradel P., Coulon J.B., Chilliard Y. (2006). Influence of grass-based diets on milk fatty acid composition and milk lipolytic system in Tarentaise and Montbe´liarde cow breeds. J. Dairy Sci..

[47] Milewski S., Ząbek K., Antoszkiewicz Z., Tański Z., Sobczak A. (2018). Impact of production season on the chemical composition and health properties of goat milk and rennet cheese. Emir. J. Food Agric..

[48] Romanzin A., Corazzin M., Piasentier E., Bovolenta S. (2013). Effect of rearing system (mountain pasture vs. indoor) of Simmental cows on milk composition and Montasio cheese characteristics. J. Dairy Res..

[49] Mele M. (2009). Designing milk fat to improve healthfulness and functional properties of dairy products: From feeding strategies to a genetic approach. Ital. J. Anim. Sci..

[50] Fieser B.G., Vanzant E.S. (2004). Interactions between supplement energy source and tall fescue hay maturity on forage utilization by beef steers. J. Anim. Sci..

[51] Ba N.X., Van Huu N., Ngoan L.D., Leddin C.M., Doyle P.T. (2008). Effects of amount of concentrate supplement on forage intake, diet digestibility and live weight gain in yellow cattle in Vietnam. Asian-Australas. J. Anim. Sci..

[52] Chen J., Liu H. (2020). Nutritional indices for assessing fatty acids: A mini-review. Int. J. Mol. Sci..

